# Readmission after index hospital discharge among patients with COVID‐19: Protocol for a systematic review and meta‐analysis

**DOI:** 10.1002/hsr2.417

**Published:** 2021-10-22

**Authors:** Abdulwahab AlSayegh, Adnan A Gassan, Ram C. Bajpai, Sara Muller, Victoria Welsh, Chun Shing Kwok, Christian D. Mallen

**Affiliations:** ^1^ School of Medicine Royal College of Surgeons in Ireland – Medical School of Bahrain Busaiteen Bahrain; ^2^ School of Medicine Keele University Keele UK; ^3^ Department of Cardiology Royal Stoke University Hospital Stoke‐on‐Trent UK

**Keywords:** coronavirus disease 2019, hospital discharge, patient outcome assessment, readmission, hospitalization

## Abstract

**Background and Aims:**

Hospital readmissions among COVID‐19 patients have increased the load on the healthcare systems and added more pressure to hospital capacity. This affects the ability to accommodate newly diagnosed COVID‐19 patients and other non‐COVID‐19 patients who require hospitalization. Therefore, this systematic review aims to understand the rates of and risk factors for hospital readmissions and all‐cause mortality among COVID‐19 patients who were hospitalized after being discharged following index hospitalization.

**Methods:**

Our systematic review protocol is registered with the International Prospective Register of Systematic Reviews (PROSPERO) (CRD42021232324) and prepared in accordance with the Preferred Reporting Items for Systematic Reviews and Meta‐Analysis Protocols (PRISMA‐P) 2015 statement. We will search MEDLINE (Ovid), EMBASE (Ovid), MedRxiv, Web of Science (Science Citation Index), ProQuest Coronavirus research database, Cochrane Covid‐19 study register, and WHO COVID‐19: Global literature on coronavirus disease will be identified from December 31, 2019, to May 31, 2021. Two investigators will independently screen titles and abstracts and select studies reporting hospital readmissions among COVID‐19 patients. Further, data extraction and risk of bias assessment will be carried out separately by these independent reviewers. We will extract data on demographics, readmissions, all‐cause mortality, emergency department visits, comorbidities, and factors associated with hospitalization among COVID‐19 patients. Random‐effect meta‐analysis will be performed if homogeneous groups of studies are found. The combined evidence will be further stratified according to important background characteristics if the data allow.

**Discussion:**

This systematic review will summarize the available epidemiological evidence regarding rates of hospital readmissions, comorbidities, and related factors among COVID‐19 patients who were readmitted after index hospitalization. A better understanding of the relationship between patient profiles and the rate of hospitalization will be helpful in the development of guidelines for patient management.

## 1 INTRODUCTION

As of September 7, 2021, the COVID‐19 pandemic has resulted in more than 222 million cases and 4.5 million deaths worldwide and will continue to have far reaching impacts on healthcare systems for many years.^1^ Most COVID‐19 cases result in mild disease that can be managed without hospitalization. However, if symptoms are severe enough, along with other underlying medical conditions, hospitalization is highly likely. Inpatient hospital readmissions due to COVID‐19 complications have been recorded but are not well understood.^2^ Hospital readmission (or rehospitalization) among COVID‐19 patients is usually defined as the inpatient hospitalization in a given follow‐up time period (such as 30 or 60 days after discharge) after discharge from index COVID‐19 related hospitalization.^2^, ^3^, ^4^, ^5^

A post‐covid syndrome has been identified and characterized as a long‐term effect with a broad range of new and existing medical conditions that are the result of COVID‐19 infection.^6^ This causes complications to different organ systems, with the major ones being the respiratory and cardiovascular systems, and ultimately results in a specific or multiorgan dysfunction.^2^, ^3^, ^4^, ^5^, ^6^ For instance, COVID‐19 pneumonia has been shown to cause lung damage reducing pulmonary function 4 months after acute infection.^7^ Inflammation from an acute infection can also cause microangiopathic thrombosis, myocarditis, cardiac arrhythmias, heart failure, and acute coronary syndrome.^8^ Other studies have also reported that COVID‐19 infection worsens the severity of existing comorbidities resulting in an increased rate of readmission.^2^, ^5^, ^6^ The major chronic conditions reported are chronic kidney disease, heart failure, and chronic obstructive pulmonary disease.^5^ Thus, COVID‐19 alters the normal physiological state of patients by new conditions or existing comorbidities, which contribute to the subsequent need for hospitalization.

The great majority of people infected with COVID‐19 survive, including those requiring hospital admission. Some patients who are discharged after hospital admission are subsequently readmitted, putting additional pressure on the availability of hospital beds. There is limited systematic evidence available on patient readmission after initial hospital discharge. Therefore, specific questions to be answered in this systematic review are as follows:

1. What are the rates of and risk factors for hospital readmission (or inpatient hospitalization), and emergency department visits among COVID‐19 patients who were discharged after index hospitalization?
2. What are the common comorbidities and other factors (demographic and clinical) associated with hospital readmission after a patient's initial COVID‐19 hospitalization and discharge?
3. What is the all‐cause mortality rate among COVID‐19 patients who were hospitalized after being discharged following index hospitalization?

## 2 METHODS

Our systematic review protocol is registered with the International Prospective Register of Systematic Reviews (PROSPERO) (CRD42021232324). This protocol is prepared using the Preferred Reporting Items for Systematic Reviews and Meta‐Analysis Protocols (PRISMA‐P) 2015 statement.^9^ This systematic review will be prepared and reported in compliance with the PRISMA 2020 statement.^10^

### 2.1 Eligibility criteria

We will include all studies of patients with COVID‐19, which report readmissions to hospital after discharge from index hospitalization. A hospital readmission can be defined as the inpatient hospitalization in a given follow‐up time period (such as 30‐ or 60‐days after discharge) after discharge from an index COVID‐19 related hospitalization.^2^, ^3^, ^4^ Studies reporting readmission after any follow‐up time will be included in this systematic review. We will include studies published in all languages. We will exclude studies if multiple publications will be identified using the same data. There are no additional exclusion criteria. COVID‐19 patients who were discharged but not readmitted will be used as the comparator, if available, to calculate the risk of, and factors associated with, hospital readmission.

### 2.2 Study design

We will include quantitative studies of any design (eg, case series, cross‐sectional, case‐control, and cohort studies). We will also include any other studies that provide required quantitative information (such as extended research letters, extended abstracts with required quantitative details). We will exclude case reports and secondary research such as reviews, systematic reviews, and evidence syntheses. However, we will check the reference list of these excluded articles for any article fitting the inclusion criteria.

### 2.3 Outcomes

#### 2.3.1 Primary outcome

- To estimate the rates of and risk factors for readmission among COVID‐19 patients who were discharged after index hospitalization.

#### 2.3.2 Secondary outcomes

- To estimate the rate of emergency department visits among COVID‐19 patients who were discharged after index hospitalization.
- To estimate mortality rates among COVID‐19 patients who were discharged after index hospitalization.
- To describe common comorbidities among COVID‐19 patients who were discharged after index hospitalization.
- To describe factors (demographic and clinical) associated with hospital readmission after a patient's initial COVID‐19 hospitalization.

### 2.4 Search strategy

Search strategies have been developed and tested through an iterative process with the help of an experienced medical information specialist. We will search MEDLINE (Ovid), EMBASE (Ovid), MedRxiv, Web of Science (Science Citation Index), ProQuest Coronavirus research database, Cochrane COVID‐19 study register, and WHO COVID‐19: Global literature on coronavirus disease between December 31, 2019 and May 31, 2021. We will also search eligible studies through the following: (a) grey literature using Google scholar and ResearchGate; and (b) checking reference lists of included studies. A draft search strategy for MEDLINE is provided in Table S1.

### 2.5 Study quality

The quality of included studies will be assessed by the National Institute of Health (NIH) quality assessment tools. The tools include items for evaluating potential flaws in study methods or implementation, including sources of bias (eg, patient selection, performance, attrition, and detection), confounding, study power, the strength of causality in the association between interventions and outcomes, and other factors.^11^ Reviewers can select “yes,” “no,” or “cannot determine/not reported/not applicable” in response to each item on the tool. For each item where “no” will be selected, reviewers are instructed to consider the potential risk of bias that could be introduced by that flaw in the study design or implementation. Cannot determine and not reported will also be noted as representing potential flaws. Quality assessment will be carried out by two reviewers independently. Discrepancies in quality appraisal scores will be resolved through discussion or referred to a third reviewer.

### 2.6 Data extraction

All search results will be downloaded from electronic databases and imported into Rayyan, an online tool, for screening to remove duplicates and data extraction.^12^ Two reviewers will independently screen titles and abstracts against selection criteria and exclude irrelevant studies by agreement. Review of full texts will be undertaken independently by two reviewers to determine eligibility. Reasons for rejecting papers at the full‐text stage will be recorded. Any differences of opinion regarding eligibility will be resolved through discussion or by consulting with a third reviewer. Studies meeting inclusion criteria will undergo data extraction by both reviewers and be independently checked (randomly 25% of included studies) by a third reviewer for accuracy and consistency. A standardized data extraction form will be designed and piloted for collecting data for analysis. At the study level, data will be extracted on the following:

- date of data extraction and data extractor
- study ID (author, year of publication)
- setting (country of origin)
- period of index hospitalization if available
- type of study
- funding sources
- age of participants (mean, SD, median, range, IQR)
- sex (counts and proportions)
- number of participants
- number of patients who were readmitted to hospital after discharge from index hospitalization
- emergency department (ED) visits after discharge from index hospitalization if reported
- number of patients who were died after discharge from index hospitalization if reported
- details of comparator group, where relevant
- description of covariates where relevant (eg, demographic information, comorbidities, type of readmission, length of hospital stay)
- measures of effect, where relevant

### 2.7 Data synthesis

Readmission rate will be calculated as the ratio between the total number of persons readmitted over the total patients who were discharged after index hospitalization and will be presented as the number of cases per 100 population. Death rate and rate of ED visits will also be calculated in a similar fashion. The reported proportions will be presented with corresponding 95% confidence intervals (CIs) using the exact binomial distribution.^13^ Meta‐analysis of proportions will be conducted if studies adequately meet the inclusion criterion and are sufficiently uniform in reporting the outcome estimates. Rates will be transformed on the double arcsine function in order to avoid variance instability and CIs exceeding the interval (0 ≤ x ≤ 1) in which proportions can be meaningfully defined. Risk of hospitalization and factors associated with hospitalization will be reported as risk ratios (RRs) with corresponding 95% CIs. A random‐effects model will be used to pool reported proportions. The residual or restricted maximum likelihood method will be used to estimate τ^2^, which produces an unbiased, non‐negative estimate of between‐study variance. This is appropriate due to the methodological heterogeneity between studies.^14^ We will investigate potential sources of heterogeneity related to both methodological and clinical characteristics of the studies using Cochran's Q test (*P* < .1 considered significant) and *I* ^*2*^ (>50% representing moderate heterogeneity) statistics.^15^ If meta‐analysis is not feasible, results will be presented narratively, and forest plots without pooling will be used to present data. Publication bias will also be assessed using funnel plot asymmetry and Egger's test for regression asymmetry (if we identify at least 10 studies).^16^ We will use the “trim and fill” analysis of Duval and Tweedie to examine the potential impact of missed or unpublished studies on the pooled estimates of the rate of readmission.^17^ Statistical analysis will be performed using Stata MP v16.1 (StataCorp, College Station, Texas) and other open‐source statistical software such as R v4.0.5 (R Foundation for Statistical Computing, Vienna, Austria) and Review Manager v5.4 (the Nordic Cochrane Centre, Rigshospitalet, Denmark).

### 2.8 Subgroup analysis

If the number of studies allows, we will analyze by subgroup according to age, sex, ethnicity, comorbidities, length of hospital stay, type of readmission (example: 14, 30, and 60 days, etc), and geographic location (eg, continent). Meta‐regression technique will also be used to explore association between certain factors (such as age, and gender) and rate of readmission if adequate number of studies will be found using the maximum likelihood method (*P* < .10 will be considered significant given the low power of these tests).^18^

### 2.9 Sensitivity analysis

Sensitivity analyses will be conducted by using influence and outlier analyses to determine the effects of certain studies on the pooled estimates of hospital readmissions.^19^ These analyses will assess how the estimated parameter of a pooled analysis would change if noisy studies were eliminated.

## 3 DISCUSSION

The synthesis of the current literature for COVID‐19 related readmissions is vital in developing mechanisms to mitigate this costly event. Several factors can play a role. The business model of different hospitals and their quality of care should be assessed for any possible associations with COVID‐19 readmission. Moreover, the heterogeneous causes such as varying comorbidities in a COVID‐19 patient result in a complex, multifaceted source for readmission.^6^, ^20^ Lastly, despite COVID‐19's presence for over a year, guidelines on post‐COVID‐19 recovery have yet to be agreed upon worldwide, and hospitals often have to employ new methods to deal with readmissions among COVID‐19 patients who initially hospitalized. Differing healthcare settings across the world may impact on the quality of care received by a patient. Essentially, most healthcare systems are divided between public and private sectors. This factor is important as during times of need, governments may call for a collaboration to enhance capacity to combat pandemics and other health emergencies.

At the core, COVID‐19's plethora of possible outcomes within a patient imposes one of the largest contributing factors to patient readmission. While many face relatively mild symptoms, unfortunately for some, life support is necessary to maintain ventilation. Consequently, a history of comorbidities such as diabetes, cardiovascular disease, or other conditions leading to immunocompromise is associated with an increased risk of readmission. Some reasons have been put forward. During the course of initial treatment, the use of intravenous fluids, anticoagulants, and glucocorticoids resulted in negative associations with COVID‐19 patient readmissions.^20^ Furthermore, when COVID‐19 patients are compared against general patients of similar personal backgrounds and clinical symptoms, they are still associated with an increased risk of readmission and death.^6^ By studying the factors associated with COVID‐19 patients, we will explore causes that may contribute to a greater risk of readmission.

There are no clear follow‐up guidelines for COVID‐19 patients post‐discharge and recovery. Instead, patients are managed on a case‐by‐case basis relying on the given symptomatic conditions to then be dealt with by a respective specialist in that field. Guidelines have now been drafted for COVID‐19 patients' initial admission,^21^, ^22^ although readmission guidelines are still lacking. This forces hospitals to employ new methods to manage patients depending upon their model of care. To alleviate this burden, this systematic review can help in formulating the first step of developing international guidance for COVID‐19 related readmissions.

## FUNDING

No specific funding was received from any bodies in the public, commercial, or not‐for‐profit sectors to conduct the work described in this manuscript.

## CONFLICT OF INTEREST

The authors declare that they have no potential conflicts of interest.

## AUTHOR CONTRIBUTIONS

Conceptualization: Ram C. Bajpai, Sara Muller, Victoria Welsh.

Data Curation: Ram C. Bajpai, Abdulwahab AlSayegh, Adnan A Gassan.

Formal Analysis: Ram C. Bajpai, Abdulwahab AlSayegh, Adnan A Gassan.

Methodology: Ram C. Bajpai, Sara Muller, and Victoria Welsh, Chun Shing Kwok, Christian D. Mallen.

Supervision: Ram C. Bajpai, Sara Muller, Victoria Welsh.

Writing—Original Draft Preparation: Abdulwahab AlSayegh, Adnan A Gassan.

Writing—Review and Editing: Ram C. Bajpai, Abdulwahab AlSayegh, Adnan A Gassan, Sara Muller, and Victoria Welsh, Chun Shing Kwok, Christian D. Mallen.

All authors have read and approved the final version of the manuscript.

Ram C. Bajpai had full access to all of the data in this study and takes complete responsibility for the integrity of the data and the accuracy of the data analysis.

## TRANSPARENCY STATEMENT

Ram C. Bajpai affirms that this manuscript is an honest, accurate, and transparent account of the study being reported; that no important aspects of the study have been omitted; and that any discrepancies from the study as planned (and, if relevant, registered) have been explained.

## ETHICAL STATEMENT

This systematic review will collect data from already published studies, which had already received ethical approval from their respective institutions.

## Supporting information


**Table S1.** Ovid MEDLINE(R) ALL <1946 to June 25, 2021>

## Data Availability

The authors confirm that the data supporting the findings of this study are available within the article and its supplementary materials.
