# Supplementary material for: Readmission after index hospital discharge among patients with COVID‐19: Protocol for a systematic review and meta‐analysis
Source: Health Sci Rep. 2021 Oct 22;4(4):e417. doi: 10.1002/hsr2.417 (PMC8532510; doi:10.1002/hsr2.417)
Supplement: Supplementary file 1 — Table S1. Ovid MEDLINE(R) ALL <1946 to June 25, 2021> [file HSR2-4-e417-s001.docx]

**Supplementary Table S1**

Ovid MEDLINE(R) ALL <1946 to June 25, 2021>

|  | Search terms |
| --- | --- |
| 1 | exp Coronavirus Infections/ |
| 2 | exp Coronavirus/ |
| 3 | (2019nCoV* or CoV2 or CoV 2).mp. |
| 4 | SARS-CoV2.mp. |
| 5 | COVID-19.mp. |
| 6 | (coronavir* or corona vir* or OC43 or NL63 or 229E or HKU1 or HCoV* or NCoV* or covid* or sarscov*).mp. |
| 7 | ((novel or new or nouveau) adj2 (CoV or Pandemi*2)).mp. |
| 8 | betacorona*.mp. |
| 9 | exp pneumonia/ and (wuhan or hubei or huanan).mp. |
| 10 | ((pneumonia or SARS or severe acute respiratory) and (wuhan or hubei or huanan)).mp. |
| 11 | ((wuhan or hubei or huanan) adj virus).mp. |
| 12 | ((wuhan or hubei or huanan) adj coronavirus).mp. |
| 13 | Wuhan coronavirus.mp. [mp=title, abstract, original title, name of substance word, subject heading word, floating sub-heading word, keyword heading word, organism supplementary concept word, protocol supplementary concept word, rare disease supplementary concept word, unique identifier, synonyms] |
| 14 | (COVID-19 or severe acute respiratory syndrome coronavirus 2).os. [supplementary concepts] |
| 15 | or/1-14 |
| 16 | patient discharge/ |
| 17 | ((patient* or hospital) adj3 discharg*).ti,ab,kf. |
| 18 | postdischarg*.ti,ab,kf. |
| 19 | ((post or follow* or after) adj3 discharg*).ti,ab,kf. |
| 20 | patient readmission/ |
| 21 | (readmit* or readmission*).ti,ab,kf. |
| 22 | (re adj (admit* or admission* or hospital*)).ti,ab,kf. |
| 23 | rehospital*.ti,ab,kf. |
| 24 | or/16-23 |
| 25 | 15 and 24 |
| 26 | limit 25 to ed=20191201-20210531 |
| 27 | limit 25 to dt=20191201-20210531 |
| 28 | 26 or 27 |
